# Preliminary Results of CitraVes™ Effects on Low Density Lipoprotein Cholesterol and Waist Circumference in Healthy Subjects after 12 Weeks: A Pilot Open-Label Study

**DOI:** 10.3390/metabo11050276

**Published:** 2021-04-27

**Authors:** Stefania Raimondo, Dragana Nikolic, Alice Conigliaro, Gianluca Giavaresi, Bruna Lo Sasso, Rosaria Vincenza Giglio, Roberta Chianetta, Mauro Manno, Samuele Raccosta, Valeria Corleone, Giovanni Ferrante, Roberto Citarrella, Manfredi Rizzo, Giacomo De Leo, Marcello Ciaccio, Giuseppe Montalto, Riccardo Alessandro

**Affiliations:** 1 Department of Biomedicine, Neuroscience and Advanced Diagnostics (Bi.N.D), Section of Biology and Genetics, University of Palermo, 90133 Palermo, Italy; dragana.nikolic@unipa.it (D.N.); alice.conigliaro@unipa.it (A.C.); giacomo.deleo@unipa.it (G.D.L.); 2 Navhetec s.r.l, Via Elvira ed Enzo Sellerio, 90141 Palermo, Italy; vacorleone@agrumariacorleone.com; 3 Department of Health Promotion Sciences Maternal and Infantile Care, Internal Medicine and Medical Specialties (PROMISE), University of Palermo, 90127 Palermo, Italy; chianetta.roberta8@gmail.com (R.C.); roberto.citarrella@unipa.it (R.C.); manfredi.rizzo@unipa.it (M.R.); giuseppe.montalto@unipa.it (G.M.); 4 IRCSS Istituto Ortopedico Rizzoli, SC Scienze e Tecnologie Chirurgiche-SS Piattaforma Scienze Omiche per Ortopedia Personalizzata, 40136 Bologna, Italy; gianluca.giavaresi@ior.it; 5 Department of Biomedicine, Neuroscience and Advanced Diagnostics (Bi.N.D), Section of Clinical Biochemistry, Clinical Molecular Medicine and Laboratory Medicine, University of Palermo, 90127 Palermo, Italy; bruna.losasso@unipa.it (B.L.S.); rosariavincenza.giglio@unipa.it (R.V.G.); marcello.ciaccio@unipa.it (M.C.); 6 Department of Laboratory Medicine, University-Hospital “P. Giaccone” of Palermo, 90127 Palermo, Italy; 7 Institute of Biophysics, National Research Council of Italy, 90146 Palermo, Italy; mauro.manno@cnr.it (M.M.); samuele.raccosta@hotmail.it (S.R.); 8 Agrumaria Corleone s.p.a., Via S. Corleone, 12-Zona Ind. Brancaccio, 90124 Palermo, Italy; gferrante@agrumariacorleone.com; 9 Institute for Biomedical Research and Innovation (IRIB), National Research Council (CNR), 90146 Palermo, Italy

**Keywords:** nutraceuticals, cardiovascular risk, LDL cholesterol, *Citrus limon* (L.) Osbeck, flavonoids

## Abstract

Appropriate monitoring and control of modifiable risk factors, such as the level of low-density lipoprotein cholesterol (LDL-C) and other types of dyslipidemia, have an important role in the prevention of cardiovascular diseases (CVD). Recently, various nutraceuticals with lipid-lowering effects have gained attention. In addition to the plant-derived bioactive compounds, recent studies suggested that plant cells are able to release small lipoproteic structures named extracellular vesicles (EVs). The interaction between EVs and mammalian cells could lead to beneficial effects through anti-inflammatory and antioxidant activities. The present study aimed to assess the safety of the new patented plant-based product citraVes™, containing extracellular vesicles (EVs) from *Citrus limon* (L.) Osbeck juice, and to investigate its ability to modulate different CV risk factors in healthy subjects. A cohort of 20 healthy volunteers was recruited in a prospective open-label study. All participants received the supplement in a spray-dried formulation at a stable dose of 1000 mg/day for 3 months. Anthropometric and hematobiochemical parameters were analyzed at the baseline and after the follow-up period of 1 and 3 months. We observed that the supplement has an effect on two key factors of cardiometabolic risk in healthy subjects. A significant change in waist circumference was found in women after 4 (85.4 [79.9, 91.0] cm, *p* < 0.005) and 12 (85.0 [80.0, 90.0] cm, *p* < 0.0005) weeks, when compared to the baseline value (87.6 [81.7, 93.6] cm). No difference was found in men (baseline: 100.3 [95.4, 105.2] cm; 4 weeks: 102.0 [95.7, 108.3] cm; 12 weeks: 100.0 [95.3, 104.7] cm). The level of LDL-C was significantly lower at 12 weeks versus 4 weeks (*p* = 0.0064). Our study evaluated, for the first time, the effects of a natural product containing plant-derived EVs on modifiable risk factors in healthy volunteers. The results support the use of EV extracts to manage cardiometabolic risk factors successfully.

## 1. Introduction

A healthy lifestyle in youth is a very effective method to address the worldwide cardiovascular disease (CVD) epidemic, the primary cause of mortality in the world with markedly increased prevalence of coronary heart disease (CHD) [1,2]. Over the past few decades, the opportunity for preventive treatments has increased along with a rising interest in dietary quality [3] supported by nutraceuticals [4]. Nutraceuticals are products containing one or more compounds derived from plants, administered in a suitable pharmaceutical form that can be consumed as dietary supplements or as part of conventional food or beverages. They have been shown to improve well-being and life quality by reducing the risk of disease development or beneficially affecting primary body functions [5].

Appropriate, timely, and continuous monitoring and control of modifiable risk factors, such as dyslipidemia and, in particular, high levels of low-density cholesterol (LDL-C), may have an important role in CVD prevention. The reduction of total cholesterol (TC) and LDL-C in preventing CVD events is supported by increasing scientific evidence [6,7]. Recently, CVD has been related to other concomitant pathologies such as osteoporosis and diabetes; moreover, these diseases share some risk factors including age, lifestyle (sedentarity, unhealthy diet, smoking, alcohol abuse), drug exposures (e.g., systemic glucocorticoids), and hypovitaminosis D [8,9,10,11].

Currently available functional foods and supplements can effectively reduce plasma LDL-C levels (from 5 to 25%), either alone or in combination [12]. Furthermore, the changes in dietary habits, including the use of functional foods enriched with phytosterol and changes in the macronutrient composition of the diet, combined with increased physical activity, are generally accepted as the most effective lifestyle changes designed to lower TC and LDL-C, increase high-density lipoprotein cholesterol (HDL-C) levels, and decrease triglyceride (TG) levels (other two strong and independent predictors of CVD) [6]. In general, it has been found that individuals at a young age with a low absolute CVD risk are more suitable candidates for being treated with these products. Furthermore, nutraceuticals also represent an alternative option to treat dyslipidemia [13,14]. These data are in agreement with the latest guidelines of lipid-level management recommending the use of innovative nutritional strategies, especially high LDL-C and TC [15]. Given that reductions in TC are usually associated with no change or mild reduction in HDL and less frequently with its increase [16,17], research into functional foods and nutraceuticals, associated with an effective physical activity (lifestyle changes), is progressing rapidly.

Recently, various nutraceuticals with lipid-lowering effects have attracted much attention [18,19], including citrus-derivatives such as citrus peel extracts, citrus flavonoids, and citrus polymethoxyflavones [20]. Furthermore, epidemiological studies have demonstrated that the intake of citrus flavonoid-containing foods is associated with a decreased incidence of CVD [21,22]. Future studies exploring the mechanism by which these active ingredients work will shed light on their potential beneficial effects on intermediate endpoints such as systemic inflammation, endothelial function, and overall CVD risk.

In addition to the properties of plants’ multiple bioactive compounds, recent studies suggest that plant cells release in the extracellular space small lipoproteic structures named extracellular vesicles (EVs). They contain molecular products of the cell including proteins, lipids, coding, and noncoding RNAs. EVs interact with mammalian cells and can be internalized leading to the modulation of different molecular pathways [23,24]. Recently, interesting anti-inflammatory properties of EVs isolated from different plant matrices such as grapes [25], broccoli [26], and ginger [27] have been highlighted. In a recent study, Berger et al. observed a protective effect of nanovesicles isolated from orange juice on obesity-associated intestinal complications [28]. We have previously isolated EVs from *Citrus limon* (L.) Osbeck juice at a laboratory scale and observed a reduction in tumor cell proliferation [29] by negatively regulating lipid metabolism [30].

The studies reported above suggest that the intake of plant-derived EVs as nutraceuticals could bring beneficial effects. For this reason, the present study aimed to assess the safety of a natural product, also containing EVs from lemon juice, and to investigate its potential in modulating different CVD risk factors in healthy subjects.

## 2. Results

### 2.1. Chemical and Biophysical Characterization of the Supplement

**Figure 1:**
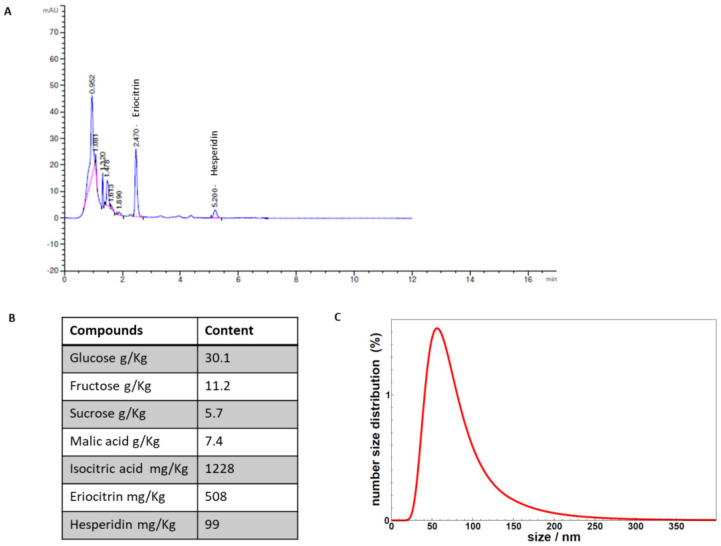
(**A**) The chromatographic analyses of the compounds identified in the supplement. (**B**) Quantitative data of the compounds quantified in the supplement. (**C**) DLS analysis of vesicles contained in the supplement.

The identification and quantification of the chemical analytes contained in the supplement are shown in Figure 1A,B. The analyses revealed the presence of the natural sugars glucose, fructose, and sucrose, the organic acids isocitric and malic, and the flavonoids eriocitrin and hesperidin. The size distribution of the vesicles contained in the supplement, determined by DLS, is shown in Figure 1C. The size of the vesicles is mainly in the range 40–100 nm, along with a tail of larger size particles still observable up to about 200 nm. DLS measurements showed the presence of vesicles with a size distribution that is not altered after maltodextrin addition and spray drying (data not shown), thus validating the sample integrity upon the overall process.

### 2.2. Effects of the Supplement on Clinical and Haematobiochemical Parameters

Demographic and tested clinical data stratified by gender at enrollment are reported in Table 1; significant differences were found between genders for quite all parameters. Before analyzing the effect of the supplement on the clinical and hematobiochemical parameters tested, we assessed whether the volunteers showed some alterations of those parameters used for the definition of the metabolic syndrome. The National Cholesterol Education/Adult Treatment Panel III (ATP III) defines metabolic syndrome as a set of risk factors considering at least three of the elements for diagnosis listed. Only one volunteer, a 60-year-old man, had high waist circumference (118 cm), systolic blood pressure (149 mmHg), and triglyceride levels (201 mg/dL) at enrollment. Thus, despite having taken the supplement, he was then excluded from the following analyses. 

Linear mixed models adjusted for age and gender were set up by considering experimental time as a fixed effect and ID_Patient as a random effect. Table 2 and Table 3 report the clinical and biochemical data considered for each experimental time. Hematological results are reported as Supplementary Table S1. No significant differences were found for clinical parameters over time, except for waist circumference (interaction experimental time–gender: F = 11.90, *p* < 0.0005) (Table 2). Significant changes in waist circumference was found in women at 4 weeks (85.4 [79.9, 91.0] cm, *p* < 0.005) and 12 weeks (85.0 [80.0, 90.0] cm, *p* < 0.0005), compared to baseline value (87.6 [81.7, 93.6] cm), while no differences were found in men.

Regarding the biochemical results, it seems that the treatment influenced glucose, triglycerides, and cholesterol metabolism in volunteers over time. The mean blood glucose values in volunteers slightly increased at 12 weeks from baseline (*p* = 0.0013), yet remaining always within the reference intervals. On the other hand, the glycosylated hemoglobin (HbA1c) decreased over time, reaching values significantly lower at 12 weeks, compared to baseline (expressed in mmol/mol: *p* = 0.0042; or in %: *p* = 0.0037). Triglycerides (interaction experimental time–gender: F = 5.25, *p* = 0.012) increased significantly for men at 4 (125.2 [83.8, 166.6], *p* = 0.005) and 12 (125.5 [101.4, 149.6], *p* < 0.0005) weeks, in comparison with baseline (100.9 [54.1, 147.7]) still remaining within the reference values, while no significant differences were found for women among experimental times (baseline: 66.0 [58.1, 73.9]; 4 weeks: 67.1 [53.1, 81.1]; and 12 weeks: 68.7 [57.8, 79.5]. Low-density lipoproteins (LDL) were significantly lower at 12 weeks versus 4 weeks (*p* < 0.0005) and baseline (*p* = 0.004). Last, alkaline phosphatase enzymes (ALP) changed significantly over time showing lower values at 4 (*p* = 0.071) and 12 (*p* = 0.030) weeks, compared to baseline.

A further analysis was carried out to evaluate the correlations between biochemical parameters that showed significant variations after 12 weeks of the supplement intake, also stratifying between women and men. Significant correlations were found in stratified analysis between ALP and glucose (r = −0.855, *p* < 0.005) for women, and between ALP and LDL (r = −0.559, *p* = 0.018) for men.

## 3. Discussion

Data from the present pilot study indicate that, in healthy subjects, 3-month consumption of the natural supplement citraVes^TM^, containing nanovesicles from *Citrus limon* L. juice, is safe and exerts positive effects reducing waist circumference and LDL cholesterol, two important CV risk factors. To our knowledge, this is the first study in which an industrial extract also containing extracellular vesicles from plants, in particular from lemons, was used. Although further studies will be necessary to discriminate their effects from those given by single lemon components and to understand its mechanism of action further, the data obtained in this study lead to encouraging future investigations. Numerous studies are showing that plant EVs play a role in the modulation of different conditions, such as inflammation and oxidative stress [25,26,27] accompanying several chronic diseases. Moreover, a recent study has shown how the consumption of orange vesicles can restore intestinal function in obesity animal models [28].

Beneficial effects on LDL-C observed in the present study are supported by numerous investigations in the literature indicating the impressive health benefits of citrus fruits, most of them even in subjects with metabolic disorders. After 6 months of lemon juice consumption (300 mL/daily), based on a mixture of juice citrus (95%) with 5% of *A. melanocarpa* extract, a significant difference in plasma cholesterol, LDL-C, and HDL-C was found in subjects with the metabolic syndrome (MetS), when compared to baseline values, although the same changes were not seen in the control group [31]. Similarly, 6 months of supplementation with bergamot juice ameliorate the lipid profile in subjects with moderate hypercholesterolemia [32]. Significant benefit on LDL-C was attributed to a high content of flavonoids in the juice of the bergamot fruit (neoeriocitrin, neohesperidin, naringin). In this study, also the values of TC and TG were reduced, while HDL-C increased. Furthermore, the quality of LDL-C changed, reducing the small, dense, and more atherogenic LDL subclasses [32], while the anthropometric parameters (body weight, waist circumference, and body mass index) slightly, but not significantly. Several clinical studies on normolipidemic and hypercholesterolemic subjects, committed to regular consumption of orange juice, have shown decreased LDL-C levels [33,34] and LDL/HDL ratio [34,35,36]. The effects of consuming red-orange juice (750 mL Sanguinea de Mombuca for 8 weeks) on the MetS risk factors were investigated in 35 healthy volunteers (man and women aged between 23 and 59 years old), divided into normal weight (*n* = 17) and overweight/obese (*n* = 12/6) [37]. A reduction in LDL-C has been reported, but no change in abdominal obesity neither in body weight, BMI, or waist circumference was observed. On the other hand, anti-inflammatory effects (reduction in C-reactive protein) and an increase in antioxidant activity have been recorded. Furthermore, long-term orange juice consumption (≥12 months, 480 mL of orange juice per day) was associated with decreased LDL-C by 18% and apolipoprotein B by 12% [35]. Additionally, TC decreased by 11%, and the LDL/HDL ratio by 12%. A similar trend was found also in the consumers with moderate hypercholesterolemia. 

The analytic characterization of citraVes^TM^ indicates the presence of the flavanones (a subgroup of flavonoid compounds) hesperidin and eriocitrin. Presumably, based on the literature evidence, the lower LDL-C seen in the present study can be attributed to citrus flavanone compounds. Naringenin and hesperetin, the aglycone of hesperidin, have been shown to inhibit in vivo acyl CoA cholesterol acyltransferase (ACAT) and microsomal transfer protein (MTP) activities, known to be responsible for cholesterol synthesis and esterification in the liver. In vitro, these two compounds were both found to reduce the levels of very low-density lipoprotein (VLDL-C) and LDL-C [38]. Other numerous studies support effective cholesterol-lowering action attributed to flavanones and vitamin C [34,36]. Hesperidin also presents several pharmacological activities, such as anti-inflammatory, antioxidation, and overall beneficial effects on the bone tissue, increasing the anabolic process and bone production [39]. Yet, eriocitrin could be responsible for the reduction of LDL-C since it is a flavanone with numerous therapeutic properties, including lipid-lowering action. In vitro studies indicate that eriocitrin can protect against diet-induced adiposity and related metabolic disorders [40]. A recent preclinical in vivo study, using rats fed with a high-fat diet, showed that eriocitrin exerts a significant antiatherosclerotic action by lowering body and organ weights, reducing lipid content and cardiac and inflammatory markers, and increasing antioxidant enzyme activities and high-density lipoprotein levels [41].

In the present study, body weight did not change significantly. This is in line with a recent meta-analysis [42] that reveals the absence of a significant difference in body weight change between grapefruits consumers and controls.

Of interest, we found a significant variation in waist circumference, although body weight did not change significantly. These findings are consistent with the results of a randomized controlled clinical trial in which overweight adults consuming grapefruit daily for 6 weeks did not show a decrease in body weight, instead significantly reduced waist circumference and LDL-C levels [43]. In addition, we found different effects on waist circumference change among gender, also supported by numerous studies in the literature indicating that it might be related to lower CV risk [44,45].

Overall, the lack of changes in body weight observed in the present study may depend on the relatively short period of observation and on the normal body weight range of the participants. Nevertheless, these results indicate that citraVes^TM^ may help to control body weight and waist circumference, in accordance with other studies [46,47].

Our findings on a variation in ALP over time might be particularly interesting since ALP has been reported to be strongly associated with the development of the MetS in healthy subjects, irrespectively of the liver dysfunction [48,49]. This link could be explained with a possible role of ALP as a marker of visceral obesity and inflammation, and ALP level might predict CVD, development of MetS, and mortality in the general population [49,50]. Our data further support the importance of the observation of ALP as a marker of metabolic risk in clinical practice, emphasizing a preventive role of the citrus supplement.

It should be mentioned that the number of clinical studies, including randomized, double-blind, placebo-controlled clinical studies, continue to grow, reporting beneficial effects of plant extracts. However, to our knowledge, this is the first study that investigates the impact of a citrus fruit extract, containing extracellular vesicles, in healthy subjects. The advantage of using EVs is that these structures, physiologically produced by plants and present in plant matrices, contain different biofunctional compounds, including lipids, proteins, and nucleic acids [24]. Moreover, considering that EVs are structures whose content is protected by a lipid membrane, their administration could increase the stability and bioavailability of many plant bioactive compounds.

Potential limitations of the present study are the limited number of participants, the short follow-up, and the absence of a control group; however, the absence of the control (placebo) group is acceptable in an open-label trial when all participants are receiving the same treatment [51] and the population included acts as their own control. Nevertheless, our study also offers some strengths, including a natural product, containing plant-derived EVs and the novelty of the study that may encourage and guide further research on this topic. The follow-up phone calls have been made during the period of the supplement consumption in order to ensure no variability in dietary habits of subjects and, actually, the small number of subjects included allowed us to monitor them closely. All the biochemical parameters were assessed in samples with blinded codes. Finally, the detailed statistical analyses that we conducted exclude the possibility that the results may have occurred randomly.

Although our findings need to be confirmed by larger trials with a longer follow-up period, they might be of important clinical impact as dyslipidemia together with visceral obesity, and the consequent cardiometabolic complications, are among the most important public health issues. Furthermore, since it has been widely described that EVs from plants deliver RNAs, including microRNAs [27,52,53], further characterization of the microRNAs in lemon EVs will provide more insight into the molecular mechanisms by which plant vesicles may mediate the biological effects already observed in this study. As recently comprehensively reviewed [54], lipoprotein(a) [Lp(a)] is an established genetic marker for CVD risk and a target for emerging therapies. However, no specific Lp(a)-lowering agent has been approved so far. Its relationship with CVD outcomes warrants further investigation that may lead to a new era in CVD prevention. Moreover, the possibility of an important role of EVs in this context should not be excluded since EVs and lipoproteins have several common biodistribution characteristics [55], although it seems that their lipid profiles are distinctly different [56]. Lastly, once the safety and the effects of the supplements have been tested on healthy volunteers, we believe that this study could be extended to subjects with metabolic disorders to prevent the development of chronic diseases, including CVDs.

## 4. Materials and Methods

### 4.1. Design of the Study

A cohort of 20 healthy volunteers (11 men and 9 women with a mean age of 48.9 ± 8.1 years old) was recruited in a pilot open-label study. All subjects were enrolled at the Unit of Diabetes and Cardiovascular Prevention, University Hospital of Palermo, Italy. Guidelines of the Strengthening the Reporting of Observational Studies in Epidemiology (STROBE) were followed for the preparation of this manuscript [57]. Written informed consent was subscribed by all participants at the enrollment. The procedures adopted in this study were in agreement with the Helsinki Declaration of 1975, and its later amendments, and were approved by the Ethics Committee of the University Hospital of Palermo, Palermo, Italy (ethical protocol code Nº 8/2017 issued on 18 September 2017, including the amendment Nº April 2019, issued on 29 April 2019). The ClinicalTrials.gov Identifier is NCT04698447. The design of the study approved by the ethical committee and registered in the ClinicalTrial.gov includes three arms: one including healthy volunteers taking the supplement and the other two arms including the subjects with the metabolic syndrome (MetS) randomly assigned, in a blinded manner, to the supplement or placebo. The healthy volunteers were treated at first in an open-label manner to test and ensure the safety of supplement compounds.

Inclusion criteria of the study were the following: (1) healthy men and women >18 years old; (2) subjects who are able to swallow; and (3) subjects who are willing to participate in the study and therefore to sign an informed consent before any study procedure. 

Exclusion criteria of the study were the following: (1) pregnancy or the willingness to become pregnant; (2) diagnosis of any of the following diseases: diabetes mellitus, dyslipidemia, hypertension, or metabolic diseases including diagnosis of the metabolic syndrome as defined by international consensus [58]; (3) severe renal or hepatic impairment; (4) known severe infections (HIV, HBV, HCV); and (5) any malignancies.

All participants were accustomed to Mediterranean dietary habits since they lived in the same city. This was also confirmed by an experienced nutritionist at the time of enrollment when the participants were interviewed regarding their lifestyle (nutrition and physical activity). During the study, they were advised to maintain their dietary habits and not to change their lifestyle. Moderate physical activity was common among healthy volunteers. The baseline characteristics of participants are shown in Table 1.

**Figure 2:**
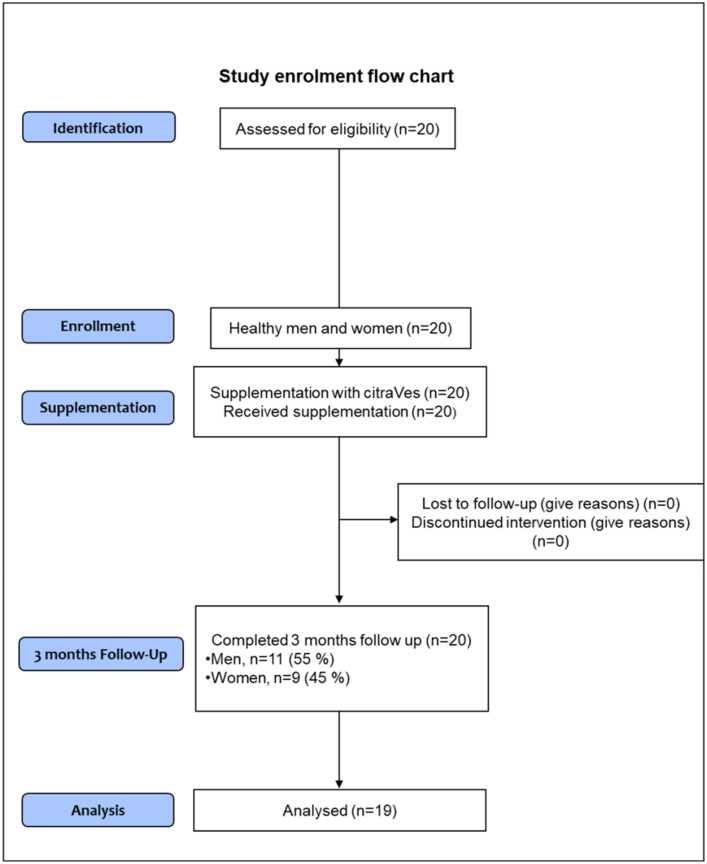
Flowchart of the study enrollment process. Due to the slightly, although not clinically significant, increase in values of waist circumference, triglycerides levels, and blood pressure during the follow-up period, one screened subject was excluded from analyses.

All participants received citraVes™ supplement in a spray-dried formulation provided by Navhetec s.r.l. *(“Nanovesicles in health and technology”*; an Innovative StartUp and Academic SpinOff of the University of Palermo, Italy). CitraVes™ is obtained from *C. limon* juice. Eligible subjects took one dose a day containing a spray-dried formulation of citraVes™, directly in the mouth and without water, to be dissolved preferably under the tongue. The supplement was given at a stable dose of 1000 mg/day for 3 months. Every month, the participants were contacted by phone to ensure compliance, including no changes in their eating and lifestyle habits, and to verify the absence of any adverse events and reinforce the adherence to the study. The site visits were performed at baseline, after 1 month, and after 3 months of the supplementation. A flowchart of the study enrollment process is shown in Figure 2.

### 4.2. Clinical Variables

At baseline, all subjects underwent a medical and physical examination to collect clinical data. Body weight and height were measured without shoes, wearing light clothing only, and using the same body scale. BMI was calculated using the standard formula (kg/m^2^). Waist circumference was measured midway between the lower rib margin and the iliac crest while the subject was in a standing position, using a nonstretchable measuring tape and to the nearest cm. The baseline blood pressure (BP) of the subject was also recorded after at least 5 min of rest in a quiet setting and a sitting position, using an automated sphygmomanometer with an adapted cuff. Blood samples were collected to analyze biochemical data. After a follow-up period of 1 and 3 months, all variables were re-evaluated: anthropometric parameters and BP were measured, and blood samples were taken to ensure the safety of the participant by monitoring renal and hepatic function.

### 4.3. Biochemical Analyses

All biochemical analyses were performed at the Department of Laboratory Medicine—University Hospital “P. Giaccone” of Palermo. Blood samples were collected after at least 8 h of overnight fasting for all laboratory tests. At baseline and after the follow-up period (1, 3 months) plasma and serum samples were gathered from each participant. HbA1c and routine clinical chemistry parameters were measured at the recruitment site immediately after sample collection. Serology test as alkaline phosphatase (ALP), alanine aminotransferase (ALT), aspartate aminotransferase (AST), high-density lipoprotein cholesterol (HDL), triglycerides (TG), low-density lipoprotein cholesterol (LDL-C), gamma-glutamyl transferase (GGT), Glycemia, Insulin, serum creatinine (sCR), total cholesterol (TC), urea, were carried out using Cobas^®^ 8000’s (Roche, Basel, Switzerland). The estimated glomerular filtration rate (eGFR) was calculated using the Chronic Kidney Disease EPIdemiology collaboration (CKD-EPI) equation expressed for the specified race, gender, and sCR in mg/dL [59]. HbA1c was measured with VARIANT II Hemoglobin (Bio-Rad). A Complete blood count (CBC) was also measured to evaluate the overall health of the participant through automated blood count on Sysmex XN-9000.

### 4.4. Determination of Chemical Analytes in the Supplement

For the determination of the chemical analytes, 2.5 g of the supplement was dissolved in 30 mL of water. The concentrations of glucose, fructose, sucrose, malic acid, and isocitric acid were determined by Icubio iMagic-M9 Automated Chemistry Analyzer. The determination of flavanones was carried out on a UHPLC Agilent 1290 Infinity System. The chromatographic analysis was performed on a C18 column (Agilent Eclipse Plus C18 2.1 × 100 mm) with an isocratic elution of 80% water, 20% acetonitrile, and 0.05% acetic acid. The flow was 0.25 mL/min for 12 min and the injection volume was 2.5 μL. The detection was carried out using a diode array detector (DAD), set at 280 nm with the acquisition of the whole spectrum from 190 nm to 400 nm. 

Flavanones were quantified based on the calibration curves for eriocitrin and hesperidin. The sample was dissolved in water and dimethylformamide filtered under a 0.45 μm membrane filter before injection.

### 4.5. Size Distribution Determined by Dynamic Light Scattering (DLS)

The samples were diluted 50 times in PBS to a final protein content below 100 μg/mL to avoid vesicle interaction and placed at 20 °C in a thermostatic cell compartment of a Brookhaven Instruments BI200-SM goniometer, equipped with a solid-state laser tuned at 633 nm. The scattered light intensity autocorrelation functions g_2_(t) were measured using a Brookhaven BI-9000 correlator (Brookhaven Instruments, Holtsville, NY, USA), and then fitted using a Schultz distribution for the diffusion coefficient D, that is, a two-parameter distribution, determined by its average and variance [60,61]. From the distribution of diffusion coefficients, one calculates the intensity-weighted distribution of hydrodynamic radii D_h_ (z-averaged size distribution) by using the Stokes–Einstein relation D = (k_B_T)/(3πηD_h_), where k_B_ is the Boltzmann constant, T is the temperature, and η is the solvent viscosity (taken as equivalent to PBS viscosity, after 50 times dilution). The number size distribution is derived by assuming that vesicles have the ideal shape of hollow spheres, in analogy to model membrane vesicles [62,63].

### 4.6. Statistical Analysis

The statistical analysis was conducted using the software R v.4.0.2 (The R Foundation, Vienna, Austria) [64]. After having verified data normality (Shapiro–Wilk test) and homogeneity of variance (Levene test), the data were analyzed with a linear mixed-effects model suitable for repeated measures, using lmer function (R package lme4 v.1.1-23) [65], with the intercept as a random effect. This model allows the heterogeneity of the outcome responses at each experimental time point and at the individual level of the volunteers. Fixed effects included in the model were those that did not show multicollinearity: age, gender, and experimental time. Pairwise comparisons were conducted on model-dependent estimated marginal means (also known as least-squares means) and adjusting the *p*-value according to Bonferroni by using emmeans function (R package emmeans v. 1.4.8) [66]. Finally, a correlation analysis was conducted to identify the relationship between the significant variations obtained in the various parameters after 12 weeks of the supplement intake. Data are reported as mean [95% CI] at a significant level of *p* < 0.05.

## 5. Conclusions

Based on the preliminary data obtained in this pilot study, the natural supplement containing EVs derived from *C. limon* juice has shown effects on two important factors of cardiometabolic risks, i.e., waist circumference and LDL-C in healthy subjects. These results encourage the use of supplements containing lemon-derived EVs to manage cardiometabolic risk factors in normolipidemic subjects. Additionally, they reinforce the currently increasing interest in nutraceuticals to prevent and delay CVDs. Further prospective studies on a larger population are required to understand the mechanisms underlying the supplement effects in the control of different CVD risk parameters and the development of new nutritional strategies.

## Figures and Tables

**Table 1:** Demographic data of healthy volunteers at enrollment (*n* = 20).

Parameter	Gender		*p*-Value	Total (*n* = 20)
	Women (*n* = 9)	Men (*n* = 11)		
**Age (years)**				
Mean [95% CI]	43 [39, 48]	54 [49, 58]	0.002	49 [45, 53]
**Age *n* (%)**				
≤50	8 (89)	4 (36)	NS	12 (60)
51–55	1 (11)	3 (27)		4 (20)
56–60	0 (0)	3 (27)		3 (15)
61–65	0 (0)	1 (9)		1 (5)
≥66	0 (0)	0 (0)		0 (0)
**Height (m)**				
Mean [95% CI]	1.68 [1.62, 1.74]	1.76 [1.73, 1.79]	0.005	1.73 [1.69, 1.76]
**Weight (kg)**				
Mean [95% CI]	68 [61, 75]	87 [79, 95]	<0.001	79 [72, 85]
**Waist circumference (cm)**				
Mean [95% CI]	88 [82, 94]	102 [96, 108]	0.001	96 [90, 101]
**BMI (kg/m^2^)**				
Mean [95% CI]	24 [22, 25]	28 [26, 30]	0.004	26 [25, 28]
**BMI *n* (%)**				
18.5–24—Healthy weight	5 (56)	1 (9)	0.031	6 (30)
25–29—Overweight	4 (44)	6 (55)		10 (50)
30–34—Class I Obesity	0 (0)	4 (36)		4 (20)
**Smoke *n* (%)**				
Yes	2 (22)	1 (9)	NS	3 (155)
No	6 (67)	10 (91)		16 (80)
ex-smokers	1 (11)	0 (0)		1 (0.05)
**Systolic Blood Pressure (mmHg)**				
Mean [95% CI]	112 [102, 121]	129 [121, 137]	0.004	121 [114, 128]
**Diastolic Blood Pressure (mmHg)**				
Mean [95% CI]	66 [59, 71]	81 [75, 87]	<0.001	74 [69, 80]

**Table 2:** Clinical parameters of healthy volunteers for each experimental time. Mean [95% CI], *n* = 19 (Women, *n* = 9 and men, *n* = 10).

Parameter	Baseline (*n* = 19)	4 Weeks (*n* = 19)	12 Weeks (*n* = 19)	*F*, *p*-Value
**Weight (kg)**				
Mean [95% CI]	77.0 [70.9, 83.1]	78.1 [71.3, 84.9]	76.6 [70.5, 82.3]	*NS*
Women	68.00 [61.5, 74.5]	67.9 [61.8, 74.0]	67.2 [61.0, 73.4]	
Men	85.1 [77.9, 92.3]	87.3 [78.7, 95.9]	85.0 [77.9, 92.1]	
**Waist circumference (cm)**				
Mean [95% CI]	94.3 [89.7, 98.9]	94.2 [88.6, 99.7]	92.9 [88.1, 97.7]	
Women	87.7 [81.7, 93.6]	85.4 [79.9, 91.0] ^1^	85.0 [80.0, 90.0] ^3^	*F* = 11.9, *p* < 0.0005
Men	100.3 [95.4, 105.2]	102.0 [95.7, 108.3]	100.0 [95.3, 104.7]	
**BMI (kg/m^2^)**				
Mean [95% CI]	25.7 [24.3, 27.2]	26.0 [24.4, 27.7]	25.6 [24.2, 27.0]	*NS*
Women	23.9 [22.3, 25.5]	23.9 [22.3, 25.5]	23.8 [22.3, 25.3]	
Men	27.4 [25.5, 29.3]	28.0 [25.7, 30.3]	27.2 [25.3, 29.1]	
**Systolic Blood Pressure (mmHg)**				
Mean [95% CI]	120 [113, 126]	118 [112, 124]	119 [113, 125]	*NS*
Women	112 [102, 121]	109 [105, 114]	112 [105, 119]	
Men	127 [120, 135]	126 [118, 134]	126 [117, 135]	
**Diastolic Blood Pressure (mmHg)**				
Mean [95% CI]	74 [68, 79]	73 [67, 78]	73 [67, 78]	*NS*
Women	66 [59, 72]	66 [62, 70]	67 [62, 73]	
Men	82 [75, 88]	79 [70, 87]	78 [69, 87]	

Pairwise comparisons among experimental times (4 weeks versus Baseline; 12 weeks versus 4 weeks; and 12 weeks versus Baseline): Waist circumference—^1^, *p* < 0.005; ^3^, *p* < 0.0005.

**Table 3:** Biochemical parameters of healthy volunteers for each experimental time. Mean [95% CI], *n* = 19.

Parameter	Baseline	4 Weeks	12 Weeks	*F*, *p*-Value	ReferenceValue *
**ALP (U/L)**	58 [52, 64]	55 [49, 61]	55 [49, 61] ^3^	*F* = 4.34, *p* = 0.020	40–129
**ALT (U/L)**	16 [13, 20]	17 [14, 21]	17 [13, 22]	*NS*	0–31
**AST (U/L)**	16 [14, 18]	15 [13, 16]	16 [13, 17]	*NS*	0–31
**γGT (mg/dL)**	17 [12, 22]	17 [13, 21]	16 [12, 21]	*NS*	5–36
**Urea (mg/dL)**	-	24 [19, 29]	34 [28, 39]	*NS*	10–50
**Creatinine (mg/dL)**	0.83 [0.76, 0.90]	0.81 [0.74, 0.88]	0.84 [0.75, 0.93]	*NS*	0.51–0.95
**eGFR (mL/min)**	97 [91, 102]	96 [91, 101]	96 [89, 103]	*NS*	>90
**Glucose (mg/dL)**	81 [77, 85]	88 [81, 96]	86 [82, 89] ^3^	*NS*	70–100
**HbA1c (mmol/mol)**	35 [33, 36]	-	33 [32, 35] ^3^	*F* = 10.51, *p* = 0.004	<40
**HbA1c (%)**	5.3 [5.2, 5.4]	-	5.2 [5.1, 5.4] ^3^	*F* = 10.87, *p* = 0.004	<5.7
**Triglycerides (mg/dL)**	84 [60, 108]	98 [73, 122] ^1^	95 [77, 114] ^3^	*F* = 6.58, *p* = 0.005	<150
**Cholesterol (mg/dL)**	168 [154, 181]	173 [158, 188]	164 [152, 176]	*NS*	<200
**HDL (mg/dL)**	53 [48, 59]	52 [47, 58]	52 [46, 58]	*NS*	>60
**LDL (mg/dL)**	97 [87, 107]	101 [88, 113]	81 [66, 95] ^2,3^	*F* = 13.17, *p* < 0.0005	<110
**IL-6 (pg/mL)**	2.5 [2.0, 3.0]	3.0 [2.0, 3.9]	2.6 [2.1, 3.1]	*NS*	<7

Pairwise comparisons among experimental times (4 weeks versus Baseline; 12 weeks versus 4 weeks; and 12 weeks veirsus Baseline): ALP—^3^, *p* < 0.05; Glucose—^3^, *p* < 0.005; HbA1c—^3^, *p* < 0.005; HbA1c %—^3^, *p* < 0.005; Triglycerides—^1^, *p* < 0.05 and ^3^, *p* < 0.05; LDL—^2^, *p* < 0.0005; ^3^, *p* < 0.005. * The reference values refer to healthy subjects according to the official guidelines.

## Data Availability

The raw data supporting the conclusions of this article will be made available by the authors upon request.

## Supplementary Materials

The following are available online at https://www.mdpi.com/article/10.3390/metabo11050276/s1, Table S1: Hematological parameters of healthy volunteers for each experimental time.
