# Supplementary material for: Preliminary Results of CitraVes™ Effects on Low Density Lipoprotein Cholesterol and Waist Circumference in Healthy Subjects after 12 Weeks: A Pilot Open-Label Study
Source: Metabolites. 2021 Apr 27;11(5):276. doi: 10.3390/metabo11050276 (PMC8145538; doi:10.3390/metabo11050276)
Supplement: Supplementary file 1 [file metabolites-11-00276-s001.zip › metabolites-1159897-supplementary.pdf]

Supplementary Material

Supplementary Data

Table S1. Hematological parameters of healthy volunteers for each experimental time. Mean [95% CI], n=19 (women, n=9; men, n=10).

Parameter	Baseline	4 weeks	12 weeks	*F, p-value*	Reference value*
**RBC (10^6 /μl)**	4.82 [4.54, 5.10]	4.88 [4.62, 5.14]	4.93 [4.62, 5.24]	*NS*	3.90 - 5.15
**Hgb (g/dl)**	13.8 [13.2 14.5]	14.0 [13.5, 14.6]	14.2 [13.4, 14.9]	*NS*	12.0 - 15.4
**Hct (%)**	41.6 [39.7, 43.6]	42.4 [40.8, 43.9]	41.4 [39.4, 43.4]	*NS*	35.5 - 45.0
**MCV (fl)**	86.8 [83.6, 89.9]	87.3 [83.6, 89.9]	84.7 [81.5, 87.8][2,3]	F=27.20, p<0.0005	78.5 - 94.5
**MCH (pg)**	28.9 [27.7, 30.0]	28.9 [27.8, 30.1]	29.0 [27.7, 30.3]	*NS*	26.3 - 32.8
**MCHC (g/dl)**	33.3 [32.8, 33.7]	33.1 [32.7, 33.6]	34.2 [33.7, 34.7][2,3]	F=12.79, p<0.0005	31.5 - 36.0
**RDW.SD (fl)**	41 [40, 43]	41 [40, 43]	40 [39, 41]	*NS*	38 - 48
**RDW.CV (%)**	13 [13, 14]	13 [13, 14]	13 [13, 14]	*NS*	11 - 15
**WBC (10^3/μl)**	6.6 [5.4, 7.7]	6.7 [6.0, 7.3]	7.0 [6.3, 7.7][3]	F=5.90, p=0.007	4.3 - 11.1
**Neutrophils (10^3/μl)**	3.9 [2.7, 5.0]	4.0 [3.3, 4.6]	4.1 [3.5, 4.7][3]	F=5.06, p=0.012	1.7 - 7.9
**Lymphocytes (10^3/μl)**	2.0 [1.7, 2.3]	2.0 [1.7, 2.3]	2.1 [1.8, 2.4]	*NS*	1.2 - 5.0
**Monocytes (10^3/μl)**	0.50 [0.43, 0.57]	0.54 [0.49, 0.59]	0.57 [0.50, 0.64]	*NS*	0.10 - 0.93
**Eosinophils (10^3/μl)**	0.15 [0.12, 0.18]	0.17 [0.13, 0.21]	0.19 [0.14, 0.25]	*NS*	0.02 - 0.60
**Basophils (10^3/μl)**	0.04 [0.03, 0.05]	0.04 [0.03, 0.05]	0.05 [0.04, 0.06]	F=5.08, p=0.012	0.00 - 0.20
**Neutrophils (%)**	56.7 [51.3, 62.3]	578.3 [53.2, 63.4]	57.7 [53.3, 62.1]	*NS*	37.5 - 77.0
**Lymphocytes (%)**	32.2 [27.5, 36.9]	30.3 [25.8, 34.9]	30.4 [26.6, 34.3]	*NS*	20.0 - 45.5
**Monocytes (%)**	7.9 [7.1, 8.7]	8.2 [7.5, 8.9]	8.4 [7.5, 9.2]	*NS*	1.6 - 9.0
**Eosinophils (%)**	2.5 [1.9, 3.1]	2. 5 [1.9, 3.0]	2.7 [2.0, 3.4]	*NS*	0.5 - 5.5
**Basophils (%)**	0.7 [0.5, 0.8]	0.7 [0.5, 0.9]	0.8 [0.6, 1.0]	*NS*	0.0 - 1.8
**IG (10^3/μl)**	0.02 [0.02, 0.02]	0.03 [0.01, 0.05]	0.03 [0.02, 0.04][2,3]	F=5.80, p=0.007	0.00 - 0.03
**IG (%)**	0.31 [0.24, 0.38]	0.37 [0.19, 0.55]	0.44 [0.31, 0.58][2,3]	F=5.69 p=0.008	0.00 - 0.05
**PLT (10^3/μl)**	217 [190, 243]	236 [213, 258]	231 [210, 252]	*NS*	170 - 400
**PDW (fl)**	15 [14, 16]	14 [14, 15]	15 [14, 16]	*NS*	9 - 14
**MPV (fl)**	11.6 [11.3, 12.0]	11.5 [11.1, 11.9]	11.5 [11.1, 11.9]	*NS*	9.2 - 12.8
**P.LCR (%)**	38.0 [35.4, 40.7]	37.4 [34.2, 40.6]	37.9 [34.8, 41.0]	*NS*	21.6 - 50.0
**PCT (%)**	0.25 [0.23, 0.27]	0.27 [0.25, 0.29][1]	0.28 [0.26, 0.30][3]	F=7.80, p=0.002	0.20 - 0.36

Pairwise comparisons among experimental times ([1], 4 weeks *versus* Baseline; [2], 12 weeks *versus* 4 weeks; and [3], 12 weeks *versus* Baseline): MCV – [2], $p<0.0005$; [3], $p<0.0005$; MCHC - [2], $p<0.0005$; [3], $p<0.0005$; WBC - [3], $p<0.05$; Neutrophils ($10^3/\mu l$) - [3], $p<0.05$; IG ($10^3/\mu l$) – [2], $p<0.05$; [3], $p<0.05$; IG % - [2], $p<0.05$; [3], $p<0.05$; PCT (%) - [1], $p<0.05$; [3], $p<0.005$. *The reference values refer to healthy subjects in according to the official guidelines.
